# Supplementary material for: Quantity and quality: Normative open-access neuroimaging databases
Source: PLoS One. 2021 Mar 11;16(3):e0248341. doi: 10.1371/journal.pone.0248341 (PMC7951909; doi:10.1371/journal.pone.0248341)
Supplement: S1 Table — N, no; Y, yes; U, unprocessed; P, processed; N/A, not applicable; NITRC, Neuroimaging Tools & Resources Collaboratory. (DOCX) [file pone.0248341.s001.docx]

**S1 Table**. **Websites, descriptor papers and download process for all presented databases**. N, no; Y, yes; U, unprocessed; P, processed; N/A, not applicable; NITRC, Neuroimaging Tools & Resources Collaboratory.

| Index | Database | Link to Database homepage | Reference for database desciptor paper | How to access | Application process | Data type |
| --- | --- | --- | --- | --- | --- | --- |
| 1 | 250 | (1) <http://hiresmri.ovgu.de/>  (2) [http://dx.doi.org/10.5061/dryad.38s74](https://doi.org/10.5061/dryad.38s74) | Lüsebrink, F., Sciarra, A., Mattern, H., Yakupov, R., & Speck, O. (2017). T1-weighted in vivo human whole brain MRI dataset with an ultrahigh isotropic resolution of 250 μm. Sci Data, 4, 170032. doi: 10.1038/sdata.2017.32 | (1) Database homepage. (2) Data download link sent after a valid email address is given. Immediate download not available. | N | U & P |
| 2 | AHEAD | [https://uvaauas.figshare.com/articles/AHEAD](https://uvaauas.figshare.com/articles/AHEAD _structural_MRI_Data/8332325)  [_structural_MRI_Data/8332325](https://uvaauas.figshare.com/articles/AHEAD _structural_MRI_Data/8332325) | Alkemade, A., Mulder, M.J., Groot, J.M., Isaacs, B.R., Van Berendonk, N., Isherwood, S., Keuken, M. C., Caan, M.W.A., Bazin, P.-L., & Forstmann, B. U. (submitted). The Amsterdam Ultra-high field adult lifespan database (AHEAD): A freely available multimodal magnetic resonance imaging database. | Data immediately downloadable from database link. | N | U |
| 3 | Age-ility | <https://www.nitrc.org/projects/age-ility/> | Karayanidis, F., Keuken, M.C., Wong, S.A., Rennie, J.L., de Hollander, G., Cooper, P.S., Fulham, W.R., Lenroot, R., Parsons, M.W., Philips, N., Michie, P.T., Forstmann, B.U. (2015). The Age-ility Project (Phase 1): Structural and functional imaging and electrophysiological data repository. Neuroimage. | Data immediately downloadable from ‘Downloads’ tab in database link. | N | U |
| 4 | ATAG | <https://www.nitrc.org/projects/atag_mri_scans> | Forstmann, B.U., Keuken, M.C., Schafer, A., Bazin, P.L., Alkemade, A., & Turner, R. (2014). Multi-modal ultra-high resolution structural 7-Tesla MRI data repository. Sci Data. | NITRC account required. Data immediately available from download tab in database link after login. | N | U |
| 5 | GSP | <https://www.neuroinfo.org/gsp/> | Holmes, A.J., Hollinshead, M.O., O'Keefe, T.M., Petrov, V.I., Fariello, G.R., Wald, L.L., Fischl, B., Rosen, B.R., Mair, R.W., Roffman, J.L., Smoller, J.W., & Buckner, R.L. (2015). Brain Genomics Superstruct Project initial data release with structural, functional, and behavioral measures. Sci Data. | Dataverse account required and access to database must be requested. A data usage agreement must be agreed to. There are no institutional barriers. Data is accessible after a valid email address is confirmed. | Y | U |
| 6 | Cam-Can | <http://www.cam-can.org/> | Taylor, J.R., Williams, N., Cusack, R., Auer, T., Shafto, M.A., Dixon, M., Tyler, L.K., Cam-Can, & Henson, R.N. (2015). The Cambridge Centre for Ageing and Neuroscience (Cam-CAN) data repository: Structural and functional MRI, MEG, and cognitive data from a cross-sectional adult lifespan sample. Neuroimage, 144, 262-269. | A data usage agreement must be agreed to. Details of institutional affiliation and a research proposal must be provided. The applicant must wait for approval to gain access to the database. | Y | U |
| 7 | DLBS | <http://fcon_1000.projects.nitrc.org/indi/retro/dlbs.html> | N/A | Data immediately downloadable from database link. | N | U |
| 8 | HCP-YA | <https://www.humanconnectome.org/study/hcp-young-adult> | Van Essen, D.C., Smith, S.M., Barch, D.M., Behrens, T.E.J., Yacoub, E., & Ugurbil, K., for the WU-Minn HCP Consortium. (2013). The WU-Minn Human Connectome Project: An overview. NeuroImage, 80, 62-79. | ConnectomeDB account is required. Valid email addresss and institution information is required to create account. Data usage terms must be agreed to. Aspera connect plugin must be downloaded and used to aid with data transfer of the large database. | Y | U & P |
| 9 | IXI | <http://brain-development.org/ixi-dataset/> | N/A | Data immediately downloadable from database link. | N | U |
| 10 | Kirby 21 | <https://www.nitrc.org/projects/multimodal> | Landman, B.A., Huang, A.J., Gifford, A., Vikram, D.S., Lim, I.A.L., Farrell, J.A.D., Bogovic, J.A., Hua, J., Chen, M., Jarso, S., Smith, S.A., Joel, S., Mori, S., Pekar, J.J., Barker, P.B., Prince, J.L., & van Zijl, P.C.M. “Multi-  Parametric Neuroimaging Reproducibility: A 3T Resource Study”. (2010). NeuroImage. | Data immediately available from ‘Downloads’ tab in database link. | N | U |
| 11 | MAASTRICHT | <https://zenodo.org/record/1206163> | Gulban, O.F., Schneider, M., Marquardt, I., Haast, R.A.M., & De Martino, F. (2018). A scalable method to improve gray matter segmentation at ultra high field MRI. PLoS One, 13, 6, e0198335. doi: 10.1371/journal.pone.0198335. | Data immediately downloadable from database link. | N | U |
| 12 | MASSIVE | <http://massive-data.org/index.html> | Froeling, M., Tax, C.M.W., Vos, S.B., Luijten, P.R., & Leemans, A. (2016). "MASSIVE" Brain Dataset: Multiple Acquisitions for Standardization of Structural Imaging Validation and Evaluation. Magnetic Resonance in Medicine, 77(5), 1797-1809. | Data immediately available from ‘Downloads’ tab in database link. | N | U |
| 13 | MSC | <https://openneuro.org/datasets/ds000224/versions/00002> | Gordon, E.M., Laumann, T.O., Gilmore, A.W., Newbold, D.J., Greene, D.J., Berg, J.J., Ortega, M., Hoyt-Drazen, C., Gratton, C., Sun, H., Hampton, J.M., Coalson, R.S., Nguyen, A.L., McDermott, K.B., Shimony, J.S., Snyder, A.Z., Schlaggar, B.L., Petersen, S.E., Nelson, S.M., & Dosenbach, N.U.F. (2017). Precision Functional Mapping of Individual Human Brains. Neuron, 95, 791–807. | Data immediately downloadable from database link. | N | U |
| 14 | MPI-CBS | <https://www.nitrc.org/frs/?group_id=606> | Tardif, C.L., Schäfer, A., Trampel, R., Villringer, A., Turner, R., & Bazin, P.L. (2016). Open Science CBS Neuroimaging Repository: Sharing ultra-high-field MR images of the brain. Neuroimage, 124, 1143-1148. | License agreement must be agreed to. Data available from ‘Downloads’ tab in database link after agreement. | N | P |
| 15 | MPI-LMBB | (1) <https://www.nitrc.org/projects/mpilmbb/>  (2) <https://www.openfmri.org/dataset/ds000221/> | Mendes, N., Oligschlaeger, S., Lauckner, M.E., Golchert, J., Huntenburg, J. M., Falkiewicz, M., Ellamil, M., Krause, S., Baczkowski, B.M., Cozatl, R., Osoianu, A., Kumral, D., Pool, J., Golz, L., Dreyer, M., Haueis, P., Jost, R., Kramarenko, Y., Engen, H., Ohrnberger, K., Gorgolewski, K.J., Farrugia, N., Babayan, A., Reiter, A., Schaare, H.L., Reinelt, J., Röbbig, J., Uhlig, M., Erbey, M., Gaebler, M., Smallwood, J., Villringer, A., Margulies, D.S. (2017). A functional connectome phenotyping dataset including cognitive state and personality measures. BioRxiv. | (1) Homepage link (2) Data download link. Data immediately available for download. | N | U |
| 16 | NKI-RS | <http://fcon_1000.projects.nitrc.org/indi/enhanced/> | Nooner, K. B., Colcombe, S. J., Tobe, R. H., Mennes, M., Benedict, M. M., Moreno, A. L., Panek, L.J., Brown, S., Zavitz, S.T., Li, Q., Sikka, S., Gutman, D., Bangaru, S., Schlachter, R.T., Kamiel, S.M., Anwar, A.R., Hinz, C.M., Kaplan, M.S., Rachlin, A.B., Adelsberg, S., Cheung, B., Khanuja, R., Yan, C., Craddock, C.C., Calhoun, V., Courtney, W., King, M., Wood, D., Cox, C.L., Kelly, A.M., Di Martino, A., Petkova, E., Reiss, P.T., Duan, N., Thomsen, D., Biswal, B., Coffey, B., Hoptman, M.J., Javitt, D.C., Pomara, N., Sidtis, J.J., Koplewicz, H.S., Castellanos, F.X., Leventhal, B.L., & Milham, M. P. (2012). The NKI-Rockland Sample: A Model for Accelerating the Pace of Discovery Science in Psychiatry. Frontiers in Neuroscience, 6, 152. | Data immediately available from the ‘Data’ tab of the database homepage. NITRC account and login required. | N | U |
| 17 | PTBP | <https://figshare.com/articles/PTBP_Nifti/1190933> | Avants, B.B., Duda, J.T., Kilroy, E., Krasileva, K., Jann, K., Kandel, B.T., Tustison, N.J., Yan, L., Jog, M., Smith, R., Wang, Y., Dapretto, M., & Wang, D.J. (2015). The pediatric template of brain perfusion. Sci Data, 2, 150003. doi: 10.1038/sdata.2015.3. | Data immediately downloadable from database link. | N | U |
| 18 | RAIDERS | <http://datasets.datalad.org/?dir=/labs/haxby/raiders> | Haxby, J.V., Guntupalli, J.S., Connolly, A.C., Halchenko, Y.O., Conroy, B.R., Gobbini, M.I., Hanke, M., & Ramadge, P.J. (2011). A Common, High-Dimensional Model of the Representational Space in Human Ventral Temporal Cortex. Neuron, 72, 404 - 416. DOI10.1016/j.neuron.2011.08.026 | Data immediately downloadable from database link. | N | U |
| 19 | SALD | <http://fcon_1000.projects.nitrc.org/indi/retro/sald.html> | Wei, D., Zhuang, K., Ai, L., Chen, Q., Yang, W., Liu, W., Wang.K., Sun, J., & Qiu, J. (2018). Structural and functional brain scans from the cross-sectional Southwest University adult lifespan dataset. Scientific Data, 5, 180134. | Data immediately downloadable from database link. | N | U |
| 20 | StudyForrest | <http://studyforrest.org/about.html> | Hanke, M., Baumgartner, F. J., Ibe, P., Kaule, F. R., Pollmann, S., Speck, O., Zinke, W., & Stadler, J. (2014). A high-resolution 7-Tesla fMRI dataset from complex natural stimulation with an audio movie. Scientific Data, 1, 1–18. | Data immediately downloadable from ‘Access’ tab in database link. | N | U |
