# Supplementary material for: Quantity and quality: Normative open-access neuroimaging databases
Source: PLoS One. 2021 Mar 11;16(3):e0248341. doi: 10.1371/journal.pone.0248341 (PMC7951909; doi:10.1371/journal.pone.0248341)
Supplement: S2 Table — ABCD; Adolescent Brain Cognitive Development study, ABIDE; Autism Brain Imaging Data Exchange, ADHD-200; Attention Deficit Hyperactivity Disorder, ADNI; Alzheimer’s Disease Neuroimaging Initiative, AIBL; Australian Imaging Biomarkers and Lifestyle Study of Aging, BRAINS; Brain Images of Normal Subjects, CMI-HBN; Child Mind Institute Healthy Brain Network, COBRE; Center for Biomedical Research Excellence, CoRR; Consortium for Reliability and Reproducibility, fBIRN; Function Biomedical Informatics Research Network, FCP; 1000 Functional Connectome Project, MIRIAD; Minimal Interval Resonance Imaging in Alzheimer’s Disease, NACC; National Alzheimer’s Coordinating Center, NCANDA; National Consortium on Alcohol and Neurodevelopment in Adolescence, OASIS; Open Access Series of Imaging Studies, PING; Pediatric Imaging, Neurocognition, and Genetics, PNC; Philadelphia Neurodevelopmental Cohort, UK; United Kingdom. + indicates that this database meets this inclusion criteria,—indicates that this database does not meet this inclusion criteria. (DOCX) [file pone.0248341.s002.docx]

**S2 Table.** **High-profile databases that do not adhere to all of our inclusion criteria.** ABCD; Adolescent Brain Cognitive Development study, ABIDE; Autism Brain Imaging Data Exchange, ADHD-200; Attention Deficit Hyperactivity Disorder, ADNI; Alzheimer’s Disease Neuroimaging Initiative, AIBL; Australian Imaging Biomarkers and Lifestyle Study of Aging, BRAINS; Brain Images of Normal Subjects, CMI-HBN; Child Mind Institute Healthy Brain Network, COBRE; Center for Biomedical Research Excellence, CoRR; Consortium for Reliability and Reproducibility, fBIRN; Function Biomedical Informatics Research Network, FCP; 1000 Functional Connectome Project, MIRIAD; Minimal Interval Resonance Imaging in Alzheimer's Disease, NACC; National Alzheimer's Coordinating Center, NCANDA; National Consortium on Alcohol and Neurodevelopment in Adolescence, OASIS; Open Access Series of Imaging Studies, PING; Pediatric Imaging, Neurocognition, and Genetics, PNC; Philadelphia Neurodevelopmental Cohort, UK; United Kingdom. + indicates that this database meets this inclusion criteria, - indicates that this database does not meet this inclusion criteria.

| **Database** | **Exclusively normative data** | **All data of similar Composition** | **Accessible to the worldwide scientific community** | **No institutional/ positional barriers** | **Free of charge** |
| --- | --- | --- | --- | --- | --- |
| ABCD | **+** | **+** | **+** | **-** | **+** |
| ABIDE | **-** | **-** | **+** | **+** | **+** |
| ADHD 200 | **-** | **-** | **+** | **+** | **+** |
| ADNI | **-** | **+** | **+** | **+** | **+** |
| AIBL | **-** | **+** | **+** | **+** | **+** |
| BRAINS | **+** | **-** | **+** | **+** | **+** |
| CMI-HBN | **+** | **-** | **+** | **+** | **+** |
| COBRE | **-** | **+** | **+** | **+** | **+** |
| CoRR | **+** | **-** | **+** | **+** | **+** |
| fBIRN | **-** | **-** | **+** | **+** | **+** |
| FCP | **+** | **-** | **+** | **+** | **+** |
| Mindboggle | **+** | **-** | **+** | **+** | **+** |
| MIRIAD | **-** | **+** | **+** | **+** | **+** |
| NACC | **-** | **-** | **+** | **+** | **+** |
| NCANDA | **+** | **+** | **+** | **-** | **+** |
| OASIS | **+** | **-** | **+** | **+** | **+** |
| OpenNeuro | **-** | **-** | **+** | **+** | **+** |
| PING | **+** | **+** | **+** | **-** | **+** |
| PNC | **+** | **+** | **+** | **-** | **+** |
| SchizConnect | **-** | **-** | **+** | **+** | **+** |
| UK Biobank | **+** | **+** | **+** | **+** | **-** |
