# Supplementary material for: Quantity and quality: Normative open-access neuroimaging databases
Source: PLoS One. 2021 Mar 11;16(3):e0248341. doi: 10.1371/journal.pone.0248341 (PMC7951909; doi:10.1371/journal.pone.0248341)
Supplement: S3 Table — All qualitative images are reported as normalized values, all quantitative images are reported as raw. SNRCC indicates the SNR of the corpus callosum and SNRCN indicates the mean SNR of both caudate nuclei. n, number of participants used for calculation; MPRAGE, magnetization prepared rapid gradient echo; MP2RAGE, magnetization prepared 2 rapid acquisition gradient echoes; ME, multiple echo; FLASH, fast low angle shot; SPACE, sampling perfection with application of optimized contrasts using different flip angle evolutions; FLAIR, fluid attenuation inversion recovery; IR, inversion recovery; TSE, turbo spin echo; TFE, turbo field echo; sb, slab; un, unprocessed; pro, processed; T1w, T1-weighted; qT1, quantitative T1 map; T2w, T2-weighted; PDw, proton density-weighted; T2*w, T2*-weighted; qT2*, quantitative T2* map; QSM, quantitative susceptibility mapping; SWI, susceptibility weighted image. (DOCX) [file pone.0248341.s003.docx]

**S3 Table.** **SNR and CNR estimations for the contrasts offered by each database**. All qualitative images are reported as normalized values, all quantitative images are reported as raw. SNR_CC_ indicates the SNR of the corpus callosum and SNR_CN_ indicates the mean SNR of both caudate nuclei. n, number of participants used for calculation; MPRAGE, magnetization prepared rapid gradient echo; MP2RAGE, magnetization prepared 2 rapid acquisition gradient echoes; ME, multiple echo; FLASH, fast low angle shot; SPACE, sampling perfection with application of optimized contrasts using different flip angle evolutions; FLAIR, fluid attenuation inversion recovery; IR, inversion recovery; TSE, turbo spin echo; TFE, turbo field echo; sb, slab; un, unprocessed; pro, processed; T1w, T1-weighted; qT1, quantitative T1 map; T2w, T2-weighted; PDw, proton density-weighted; T2*w, T2*-weighted; qT2*, quantitative T2* map; QSM, quantitative susceptibility mapping; SWI, susceptibility weighted image.

| Database | Sequence | Contrast | SNR_CC_ (± SEM) | SNR_CN_ (± SEM) | CNR (± SEM) | n | Resolution (mm) |
| --- | --- | --- | --- | --- | --- | --- | --- |
| 250 | MPRAGE | T1w-un | 292.3 ± 15.0 | 198.3 ± 14.6 | 93.5 ± 6.7 | 1 | 0.25 x 0.25 x 0.25 |
|  | MPRAGE | T1w-pro | 570.4 ± 123.5 | 368.0 ± 53.5 | 93.7 ± 14.6 | 1 | 0.25 x 0.25 x 0.25 |
| Age-ility | MPRAGE | T1w | 31.4 ± 2.0 | 20.4 ± 2.0 | 5.8 ± 0.9 | 5 | 1 x 1 x 1 |
| AHEAD | MP2RAGEME | T1w | 83.4 ± 6.5 | 39.5 ± 1.3 | 28.5 ± 1.3 | 15 | 0.64 x 0.64 x 0.7 |
|  | MP2RAGEME | qT1 | 24.2 ± 2.4 | 19.6 ± 1.1 | 6.0 ± 0.6 | 15 | 0.64 x 0.64 x 0.7 |
|  | MP2RAGEME | QSM | 10.0 ± 2.6 | 16.0 ± 1.5 | 8.7 ± 1.8 | 15 | 0.64 x 0.64 x 0.7 |
|  | MP2RAGEME | PDw | 97.5 ± 6.2 | 29.5 ± 2.4 | 1.7 ± 0.5 | 15 | 0.64 x 0.64 x 0.7 |
|  | MP2RAGEME | T2*w | 77.7 ± 12.0 | 25.3 ± 2.6 | 0.3 ± 0.5 | 15 | 0.64 x 0.64 x 0.7 |
|  | MP2RAGEME | qT2* | 10.6 ± 0.6 | 6.3 ± 0.4 | 0.3 ± 0.1 | 15 | 0.64 x 0.64 x 0.7 |
|  | MP2RAGEME | T1w-sb | 157.1 ± 7.5 | 103.1 ± 5.7 | 37.4 ± 1.3 | 15 | 0.5 x 0.5 x 0.5 |
|  | MP2RAGEME | qT1-sb | 15.4 ± 0.7 | 14.4 ± 0.7 | 4.3 ± 0.2 | 15 | 0.5 x 0.5 x 0.5 |
|  | MP2RAGEME | PDw-sb | 147.8 ± 8.2 | 79.5 ± 6.4 | 8.3 ± 2.5 | 15 | 0.5 x 0.5 x 0.5 |
|  | MP2RAGEME | T2*w-sb | 138.1 ± 21.1 | 83.8 ± 9.8 | 5.0 ± 1.7 | 15 | 0.5 x 0.5 x 0.5 |
|  | MP2RAGEME | qT2*-sb | 8 ± 0.3 | 6.2 ± 0.4 | 0.8 ± 0.2 | 15 | 0.5 x 0.5 x 0.5 |
| ATAG | MP2RAGE | T1w | 118.6 ± 6.5 | 29.6 ± 1.4 | 19.6 ± 0.7 | 15 | 0.7 x 0.7 x 0.7 |
|  | MP2RAGE | qT1 | 23.7 ± 0.9 | 17.8 ± 0.6 | 6.2 ± 0.2 | 15 | 0.7 x 0.7 x 0.7 |
|  | ME-3D-FLASH | T2*w | 128.5 ± 11.4 | 85.5 ± 10.6 | 2.8 ± 1.9 | 15 | 0.5 x 0.5 x 0.5 |
|  | MP2RAGE | T1w-sb | 146.2 ± 9.1 | 47.9 ± 2.1 | 23.3 ± 1.3 | 15 | 0.6 x 0.6 x 0.6 |
|  | MP2RAGE | qT1-sb | 23.8 ± 1.1 | 19.6 ± 0.7 | 4.9 ± 0.3 | 15 | 0.6 x 0.6 x 0.6 |
| Cam-Can | MPRAGE | T1w | 32.8 ± 2.7 | 24.1 ± 1.2 | 6.1 ± 0.5 | 15 | 1 x 1 x 1 |
|  | SPACE | T2w | 11.8 ± 1.0 | 14.3 ± 0.7 | 2.7 ± 0.2 | 15 | 1 x 1 x 1 |
| GSP | MEMPRAGE | T1w | 15.8 ± 0.8 | 8.8 ± 0.7 | 3.6 ± 0.2 | 5 | 1.2 x 1.2 x 1.2 |
| DLBS | MPRAGE | T1w | 38.3 ± 4.2 | 19.4 ± 2.7 | 4.8 ± 0.7 | 15 | 1 x 1 x 1 |
| HCPYA | MPRAGE | T1w-un | 54.7 ± 5.6 | 40.4 ± 1.8 | 9.1 ± 1.3 | 5 | 0.7 x 0.7 x 0.7 |
|  | SPACE | T2w-un | 26.7 ± 2.6 | 37.7 ± 3 | 11.5 ± 0.3 | 5 | 0.7 x 0.7 x 0.7 |
|  | MPRAGE | T1w-pro | 87.8 ± 13.0 | 72.9 ± 12.9 | 16.3 ± 2.8 | 5 | 0.7 x 0.7 x 0.7 |
|  | SPACE | T2w-pro | 32.8 ± 2.1 | 57.1 ± 8.5 | 14.6 ± 3.1 | 5 | 0.7 x 0.7 x 0.7 |
| IXI | - | T1w | 58.0 ± 3.6 | 33.7 ± 1.8 | 4.2 ± 0.4 | 15 | 0.94 x 0.94 x 1.2 |
|  | - | T2w | 15.5 ± 1.4 | 17.7 ± 1 | 4.0 ± 0.2 | 15 | 0.9 x 0.9 x 1.2 |
|  | - | PDw | 58.0 ± 3.9 | 35.1 ± 2 | 8.3 ± 0.4 | 15 | 0.9 x 0.9 x 1.2 |
| Kirby 21 | MPRAGE | T1w | 34.2 ± 1.8 | 16.7 ± 1.1 | 6.9 ± 0.6 | 5 | 1 x 1 x 1.2 |
|  | FLAIR | FLAIR | 14.0 ± 1.6 | 8.0 ± 1.1 | 1.7 ± 0.3 | 5 | 1.1 x 1.1 x 1.1 |
| MAASTRICHT | MPRAGE | T1w | 96.9 ± 2.2 | 36.6 ± 3.3 | 18.5 ± 2.1 | 5 | 0.7 x 0.7 x 0.7 |
|  | MPRAGE | PDw | 129.0 ± 11.0 | 85.3 ± 22.1 | 2.6 ± 0.9 | 5 | 0.7 x 0.7 x 0.7 |
|  | MPRAGE | T2*w | 55.3 ± 8.9 | 37 ± 5 | 4.0 ± 1.6 | 5 | 0.7 x 0.7 x 0.7 |
| MPI-CBS | MP2RAGE | T1w | 271.3 ± 31.7 | 93.1 ± 15.9 | 42.4 ± 5.4 | 5 | 0.5 x 0.5 x 0.5 |
|  | MP2RAGE | qT1 | 24.7 ± 3.2 | 18.9 ± 2.9 | 5 ± 0.7 | 5 | 0.5 x 0.5 x 0.5 |
|  | ME-FLASH | T2*w | 129.6 ± 24.1 | 83.9 ± 22 | 6.4 ± 2.5 | 5 | 0.5 x 0.5 x 0.5 |
|  | ME-FLASH | qT2* | 9.1 ± 0.6 | 4.7 ± 0.8 | 0.1 ± 0.2 | 5 | 0.5 x 0.5 x 0.5 |
| MPI-LMBB | MP2RAGE | T1w | 26.6 ± 1.3 | 12.7 ± 0.3 | 6.4 ± 0.3 | 15 | 1 x 1 x 1 |
|  | MP2RAGE | qT1 | 17.2 ± 0.8 | 17 ± 0.4 | 5.9 ± 0.2 | 15 | 1 x 1 x 1 |
|  | - | FLAIR | 81.7 ± 7.0 | 82 ± 5 .0 | 16.5 ± 1.4 | 10 | 0.49 x 0.49 x 1 |
| MASSIVE | 3D-TFE | T1w | 17.2 ± 1.2 | 10.6 ± 0.3 | 6.4 ± 0.3 | 1 | 1 x 1 x 1 |
|  | 3D-TSE | T2w | 21.9 ± 2.0 | 13.8 ± 1.8 | 2.2 ± 0.4 | 1 | 1 x 1 x 1 |
|  | 3D-IR-TSE | FLAIR | 15.9 ± 1.4 | 13 ± 1.2 | 2.6 ± 0.2 | 1 | 1 x 1 x 1 |
| MSC | - | T1w | 40.0 ± 2.0 | 26.6 ± 1.3 | 9.3 ± 0.2 | 5 | 0.8 x 0.8 x 0.8 |
|  | - | T2w | 16.0 ± 1.5 | 23 ± 2.3 | 5.2 ± 0.7 | 5 | 0.8 x 0.8 x 0.8 |
| NKI-RS | MPRAGE | T1w | 44.5 ± 2.7 | 28.1 ± 1.0 | 7.8 ± 0.4 | 15 | 1 x 1 x 1 |
| PTBP | MPRAGE | T1w | 43.6 ± 3.6 | 28.2 ± 1.8 | 9.2 ± 0.7 | 5 | 1 x 1 x 1 |
| RAIDERS | MPRAGE | T1w | 28.9 ± 3.8 | 16.9 ± 1.6 | 8.8 ± 0.9 | 5 | 0.938 x 0.938 x 1 |
| SALD | MPRAGE | T1w | 35.1 ± 2.0 | 23.9 ± 0.8 | 6.0 ± 0.2 | 15 | 1 x 1 x 1 |
| StudyForrest | 3D-TFE | T1w | 75.2 ± 7.5 | 43.5 ± 4.1 | 18.5 ± 1.1 | 5 | 0.67 x 0.67 x 0.7 |
|  | 3D-TSE | T2w | 51.6 ± 7.8 | 66.0 ± 3.0 | 10.2 ± 1.0 | 5 | 0.67 x 0.67 x 0.7 |
|  | 3D Presto FFE | SWI | 72.0 ± 11.2 | 40.3 ± 1.9 | 1.2 ± 1.2 | 5 | 0.43 x 0.43 x 0.35 |
