# Supplementary figures and images for: Quantity and quality: Normative open-access neuroimaging databases
Source: PLoS One. 2021 Mar 11;16(3):e0248341. doi: 10.1371/journal.pone.0248341 (PMC7951909; doi:10.1371/journal.pone.0248341)

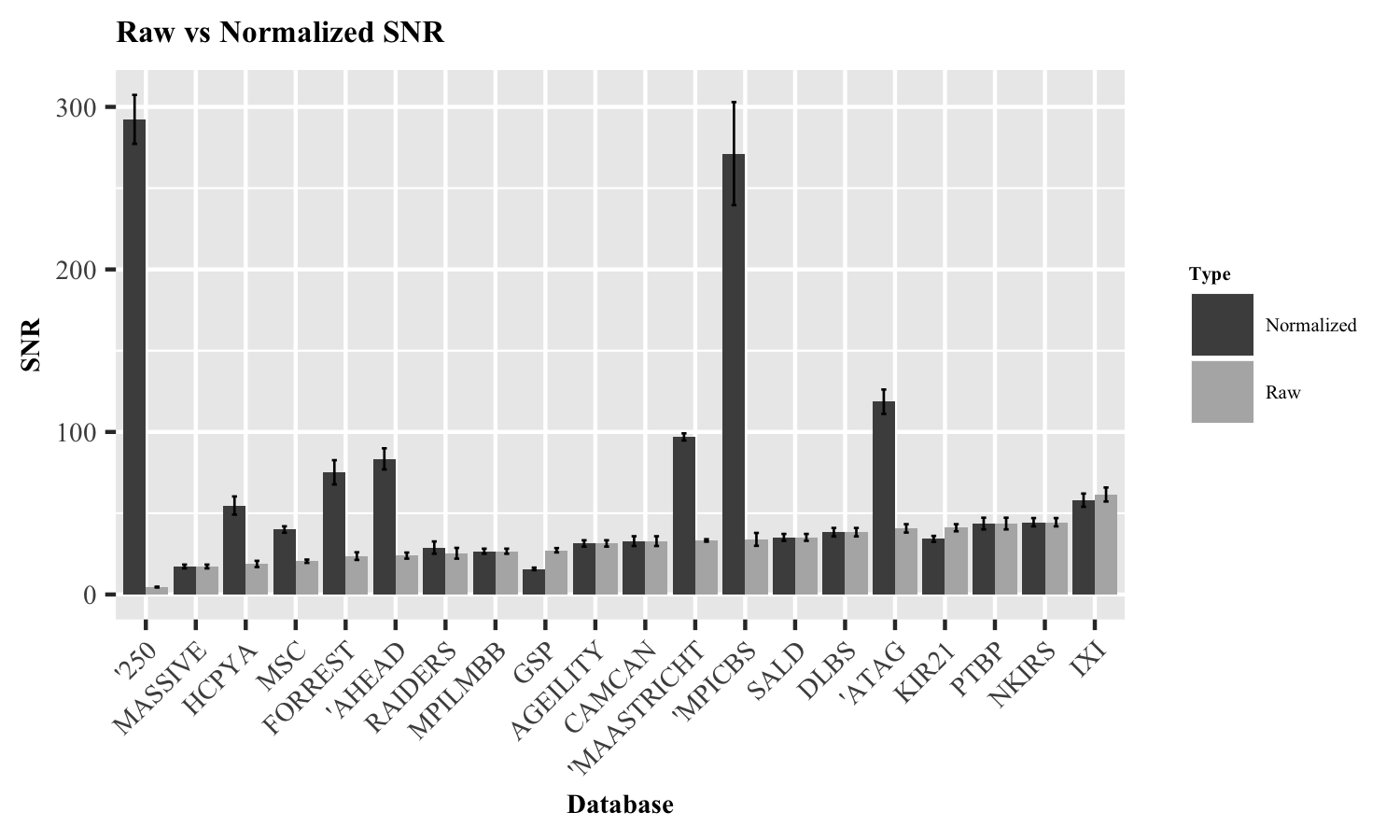

Supplement: S1 Fig — Values are ordered from lowest to highest, based on the raw SNR measurements. Error bars indicate standard error of the mean. (TIFF) [file pone.0248341.s004.tiff]
