# Supplementary material for: Quantity and quality: Normative open-access neuroimaging databases
Source: PLoS One. 2021 Mar 11;16(3):e0248341. doi: 10.1371/journal.pone.0248341 (PMC7951909; doi:10.1371/journal.pone.0248341)
Supplement: Attachment — Submitted filename: response_to_reviewer_plosone_QQ.docx [file pone.0248341.s006.docx]

**We thank the editor and reviewers for their critical appraisal of our work. In response to their questions, we have added new validation analyses, more detailed discussions of the results and further clarifications. We provide below a point-by-point response (in blue) along with the changes made to the manuscript (in italics).**

**Editor’s comments:**

**(i) I recommend you to clearly state the need for SNR and CNR metrics in these datasets. You mentioned briefly in the introduction section that cortical surface mapping is dependent on SNR and CNR, however I think you need to cite references and review thoroughly any analysis bias has been induced due to SNR and CNR variations in the literature.**

We agree with the editor that this needs to be made clearer in the article. We clarified the importance of SNR and CNR measurements in then introduction (page 2, line 67):

*In short, the SNR infers the propensity of an MR image to delineate brain structures and detect pathology (Michaely et al., 2007). By providing these estimates for each database, we are giving a quantitative measure of two dimensions; image quality (SNR) and contrast (CNR). An increase in these quantitative measures improve the qualitative ability of e.g., manual or automatic parcellation. The CNR gives a valuable inference on the ability to spatially resolve detail in an image. Therefore, using different databases with varying CNRs may result in different outcomes depending on the reason they are being used (e.g., segmentation, volumetric measurements, delineation of cortical folding). SNR inherently provides an estimate of the noise level in a structure or image and higher image quality is both quantitively and qualitatively useful. Of course, the SNR is often used as a trade-off parameter to gain improvements in another aspect of the imaging method such as resolution, scan time, field of view (FOV) and indirectly, sample size. For example, a database with a low CNR and a large sample size may not be pragmatic to use for the segmentation of subcortical nuclei but would provide accurate volumetric whole brain estimates of a population. Conversely, a database with a high CNR and small sample size may not be able to provide reliable information at a population level but may deliver an insight into the substructure of a single region. Thus, larger databases with vast and multimodal data of each individual have already provided population-level information on cortical arrangement as well as the impact of genetics and the environment on the human brain (Glasser et al., 2016; Blesa et al., 2016; Strike et al., 2019; Cheng et al., 2020; Bingjiang et al., 2020; Bischof & Park, 2015) which would not be possible in smaller databases.*

**(ii) there are many ways to calculate SNR and CNR, I recommend your discuss this in your discussion section.**

We thank the editor for this comment and agree that there is indeed an array of methods to calculate the SNR of anatomical MR images. As you have suggested, we have added the following to the discussion section of the article (page 18, line 577):

*It should be stressed that there are a variety of methods to calculate both the SNR and CNR of structural MR images, note that most of these methods are not applicable to all situations. For SNR estimation, there are two other prominent methods used in the field. The first involves measuring the SNR of the region of interest (ROI) within the brain and dividing it by the SNR of the background of the image outside of the brain. The second involves measuring only the mean signal of the ROI inside the brain and dividing it by the standard deviation of a region outside of the brain. A commonality in both of these methods is that they assume that measuring an area outside of the brain captures only the noise induced by the MR scanner itself. One reason we opted for the method used here is that due to inhomogeneities in the magnetic field of each scanner and differences in the spatial distribution of noise (Sodickson & Manning, 1997; Pruessmann et al., 1999), the area of the background image chosen for the measurement of noise could differ significantly between sites and sequences. Of course, our method does not remove the problem of bias, but as this bias is the same across all of the images measured here, we believe the comparison is fair. Regardless of the method used for the measurement of the SNR, the most important requirement for an objective comparison is that the method used is consistent across all data.*

**Reviewer 1’s comments:**

**The authors have presented an analysis on the SNR and CNR for 20 publically available MRI databases and also compared the age related changes in SNR and CNR. The manuscript is organized well and is informative. Following are some concerns/comments:

Major:
1. Some of the databases have large number of images, and thus taking 5 sample randomly may not be a true reflection of the whole population. Can authors randomly take separate 5 samples from each database (where available) and check if the same analysis still holds?**

We agree with the reviewer that five participants in some cases are a very small proportion of the overall database sample and therefore may not represent the database as a whole. To address this, we have performed the suggested validation experiment and added the following to the methods (page 6, line 222) and results (page 12, line 376) section:

*To address the issue of reliability when taking a small subpopulation from large samples, we re-ran some of the SNR and CNR analysis with a different sample from the databases. 5 or 15 (if they included large age-ranges) additional samples were taken from each database that allowed it (dependent on the original sample size) and SNR measurements were calculated again from their T1w images for comparison against the original sample. Of the 20 databases included in this article, 17 had a sample size large enough for us to take additional measurements. SNR measurements were taken from the left caudate nucleus, right caudate nucleus and corpus callosum of 164 separate T1w images.*

*To investigate the similarity of the first sample of measurements to the second sample, a Bayesian ANOVA was used to provide evidence for or against the null hypothesis (that these samples were taken from the same distribution). The Bayes Factors (BF) resulting from this analysis for each structure are as follows: corpus callosum SNR BF = 0.139, caudate nuclei SNR BF = 0.129, and the CNR BF = 0.224. Based on Jeffreys (1961), this provides substantial evidence for the null hypothesis, that both samples from each database come from the same distribution. This shows that our sample-based method is reproducible across samples of the databases. Although it would ideally be best to manually segment the CC and CN in each subject, the simplified approach we take here provides a good trade-off between accuracy and manageability given the large of amount of manual delineation that had to be done in the original (665) and the second (164) sample.*

**2. It appears that the SNR was calculated using 3x3x3 voxel only? If only a single ROI of 3x3x3 was used for calculation then it is prone to large errors due to a few pixels offset in the location of the ROI. A better estimate might be to perform a moving window averaging of 3x3x3 mask over a larger ROI (CC, CN)**

As discussed above, due to the large number of subjects sampled, manual delineations of larger ROIs are very challenging. Automated segmentations may also be biased due to differences in the SNR and CNR we wish to measure. In addition, there is a large variability in voxel dimensions across databases, making it in some cases quite challenging to find 3x3x3 voxel cube fully inside the structures tested (CN particularly). Thus, to avoid biases against coarser databases, we opted for this simple point-wise estimate based on 27 voxels inside each structure, ensuring as much tissue homogeneity as possible. As demonstrated by the validation study above, this estimate is consistent within each database, thus giving a reasonable estimate of its quality. We added the following comments in the discussion (page 18, line 589).

*To signify that this method was indeed reliable within the databases, we ran the validation study on the separate T1w images. The reproducibility of the estimates that we took indicate that the methods holds as a consistent measurement of SNR.*

**Minor:
1. Fig. 1, please indicate CC, RCN, LCN regions in the images.**

We thank the rater for bringing this to our attention, we have uploaded a new one which hopefully solves this issue, see below:

**2. PRISMA abbreviation?**

We thank the reviewer for pointing this out and we have added the meaning of the abbreviation PRISMA to the main text (page 6, line 234):

*Preferred Reporting Items for Systematic Reviews and Meta-Analyses.*

**3. It might be better to perform separate analysis of scan time vs SNR for 3T and 7T. Since SNR depends on both the field strength and scan time.**

We thank the reviewer for this suggestion and agree that this would provide a less biased comparisons. We have adapted the graph and the analysis to reflect this. We have provided a new image showing the correlation within 3T and 7T separately and adapted the results section (page 13, line 407):

*Fig 8A shows the relationship between scan time and the normalized SNR for both 3T and 7T scanners separately. It can be seen that there is a significant positive correlation within the 3T data, and the 7T data displays the same trend but does not show significance.*

**4. Which data supports the following line in the conclusion.
“The benefit of large-scale imaging databases for creating general maps of cortical organization and providing both phenotypic and genetic comparisons across populations is clear.”**

The reviewer is correct that we failed to provide clear evidence for this statement in the conclusion. We have addressed this in the introduction by adding references for this point (page 3, line 86). See below and our response to the editor’s comment (i):

*Thus, larger databases with vast and multimodal data of each individual have already provided population-level information on cortical arrangement as well as the impact of genetics and the environment on the human brain (Glasser et al., 2016; Blesa et al., 2016; Strike et al., 2019; Cheng et al., 2020; Bingjiang et al., 2020; Bischof & Park, 2015) which would not be possible in smaller, databases.*

**5. Another line in conclusion “In particular for the human subcortex ….. does not provide the required resolution and SNR for small nuclei.” There was no analysis on the resolution in this study?**

We thank the reviewer for pointing this out. We did not conduct any analyses on spatial resolution and the visualization of small brain structures. We should have been clearer that we were referring to previous work and the need for high signal and resolution to accurately observe the structure of the subcortex. To make this clearer we have added the following to the introduction (page 3, line 98):

*UHF MRI can provide reduced partial volume effects due to increased spatial resolution, allowing for the visualization of finer anatomical detail (Lüsebrink et al., 2013; Federau and Gallichan 2016). Historically, the lack of signal and contrast within the deep brain is the reason for the only recent development of subcortical maps in vivo (Johansen-Berg et al., 2013; Keuken et al., 2015). UHF MRI and its accompanying increase in SNR and contrast capacity will aid in the understanding of the structure of these deeper structures.*
